# 

**Published:** 1893-12

**Authors:** 


### Plymouth Medical Society.

### The Library of the Bristol Medico-Chirurgical Society.

At the Annual Meeting held on October 11th, 1893, Mr. L. M. Griffiths, the Honorary Librarian, read the following Report:—

During the last twelve months considerable changes in connection with our Library have taken place. The proposed junction with the Library of the Medical School—or, to speak more correctly, with the Medical Library of the University College—has been made. The details of the arrangement are contained in the code, a copy of which our Secretary sent to each member with the notice of this meeting.

After a comfortable stay of two and a-half years in the club house of the Literary and Philosophic Club, we were able on the 1st July of this year to avail ourselves of more spacious quarters in the Medical Wing of the University College. The new arrangement promises to work well. It is hoped that you will consider that your interests, which you entrusted to three representatives of the Society, have been sufficiently safeguarded in the event of future emergencies.

In addition to our own books, those of the Medical Library of the University College can now be used by members of this Society. A clerk is in charge of the Library, and, according to agreement, is always present personally or by deputy in the Library, and ready to give information to members concerning the contents of the Library. Arrangements for cataloguing are in progress, and these when completed will offer every facility to readers. It will be some time before the cataloguing is in order, as it is a matter of considerable and tedious labour, and the work of moving and arranging our possessions has taken nearly all the time up to the present.

Since the last Annual Meeting 731 volumes have been added to the Library, which now consists, including 381 duplicates, of 3,295 volumes. Amongst the additions will be found many recently published works. Duplicates, which are placed in the reading room downstairs, may under certain conditions be taken out on loan.

The donors during the twelve months have been: Mr. W. M. Barclay, Mr. T. G. L. Baretti, Dr. J. Beddoe, British Medical Association, Mr. A. Carr, Mrs. J. P. Challacombe, Mr. F. R. Cross, Dr. T. Fisher, Mr. W. H. Harsant, Mr. F. P. Lansdown, Mr. J. K. Morgan, Dr. A. W. C. Peskett, Mr. C. F. Pickering, Dr. W. E. Pountney, Dr. B. M. H. Rogers, the Royal Medical and Chirurgical Society, Sir James Sawyer, Dr. J. E. Shaw, Dr. Shingleton Smith, the Staff of St. Thomas's Hospital, the Surgeon-General United States Army, and Mr. A. J. Turner.

Neither section of the Library grows as rapidly as it ought. Both suffer from a scarcity of donors. When your Library was started in the year 1891 some members readily made donations of money and books. But amongst those who gave and those who did not give, there were many who desired to postpone anything in the way of substantial gifts till a larger library scheme was in operation. These contributions have not yet been received in anything like the measure that was to be expected. Doubtless, members are waiting till the opening of the Session, when the urgent medical life of the neighbourhood renews its vigour.

The Library Committee will, I am sure, be glad to receive gifts of suitable books. These, in a library, are of a very varied character; in fact, it may be said that anything is acceptable. In the present state of their finances the Library Committee feel that for making up sets of back periodicals they ought to rely almost entirely upon gifts. To those who prefer giving money, I may point out that a donation of ten guineas constitutes life-membership.

---

*The following donations have been received since the publication of the list in June:*

*November 30th*, 1893.

| | |
|---|---|
| J. W. Arrowsmith (1) | 2 volumes. |
| John Ewens (2) | 1 volume. |
| T. Fisher, M.D. (3) | 3 volumes. |
| F. P. Lansdown (4) | 1 volume. |
| Arthur B. Prowse, M.D. (5) | 1 ,, |
| R. Shingleton Smith, M.D. (6) | 19 volumes. |
| F. W. Stoddart (7) | 1 volume. |
| The Council of University College, Bristol (8) | 1 ,, |
| Algernon Warren (9) | 10 volumes. |

A Photograph of Rembrandt's "Anatomical Lesson" has been given by Mr. James Fawn. Messrs. George's Sons have presented some Medical and Anatomical Plates.

### TENTH LIST OF BOOKS.

The figures in brackets refer to the figures after the names of the donors, and show by whom the volumes were presented. The books to which no such figures are attached have either been bought from the Library Fund or received through the *Journal.*

---

**Anderson, T. M.**... *A Treatise on Diseases of the Skin* ... ... ... ... ... ... 1887

**Andral, G.** ... ... *Précis d'Anatomie pathologique* ... (9) Tomes I., II. 1829

**Ashby and G. A. Wright, H.** *The Diseases of Children* ... ... 2nd Ed. 1892

**Barker, A. E. J.**... *Surgical Operations* ... ... ... ... ... ... ... ... 1887

**Béclard, P.-A.** ... *Élémens d'Anatomie générale* ... ... ... (9) 2nd Ed. 1827

**Brodie, C. G.** ... *Dissections Illustrated* ... ... ... ... Part II. [1893]

**Bruce, A.** ... ... *Illustrations of the Mid and Hind Brain* ... ... ... 1892

**Bryant, T.** ... ... *The Diseases of the Breast* ... ... ... ... ... ... ... 1887

,, ... ... *Colotomy* ... ... ... ... ... ... ... ... ... ... ... ... 1890

**Burckhardt and E. H. Fenwick, E.** *Atlas of Electric Cystoscopy* 1893

**Casper, J. L.** ... *Forensic Medicine.* 3rd Ed. (Tr. by G. W. Balfour) Vol. IV. 1865

**Charcot, J. M.** ... *Localisation of Cerebral and Spinal Diseases.* (Tr. and Ed. by W. B. Hadden) ... ... ... ... ... ... 1883

,, ... *Diseases of the Nervous System.* (Tr. by T. Savill) Vol. III. 1889

**Churchill, J. F.**... *The Stœchiological Cure of Consumption.* 3rd Ed. 1893

**Clarke, J. J.** ... *Cancer, Sarcoma, &c., in relation to Sporozoa* ... ... 1893

**Clemow, F.** ... *The Cholera Epidemic of* 1892 *in the Russian Empire* ... 1893

**Cohnheim, J.** ... *Lectures on General Pathology.* 2nd Ed. (Tr. by A. B. McKee.) 3 vols. ... ... ... ... ... ... 1889-90

**Cotterell, E.** ... *Syphilis* ... ... ... ... ... ... ... ... ... ... ... ... 1893

**Craig and W. Maule, J.** *Medical Tracts for the Times.* 1st Series... (1) 1886

**Creighton, C.** ... *A History of Epidemics in Britain* (A.D. 664—1666) ... 1891

**Cripps, H.** ... ... *Diseases of the Rectum and Anus* ... ... ... 2nd Ed. 1890

**Dalby, Sir W. B.** *Diseases and Injuries of the Ear* ... ... ... 4th Ed. 1893

**Davidson, A.** ... *Geographical Pathology.* 2 vols. ... ... ... ... ... 1892

*Dermatology, Selected Monographs on* ... ... ... ... ... ... ... ... 1893

**Donkin, H. B.** ... *The Diseases of Childhood (Medical)* ... ... ... ... 1893

**Doran, A. H. G.**... *Handbook of Gynæcological Operations* ... ... ... ... 1887

**Duchenne, Dr.** ... *Selections from Clinical Works.* (Tr. and Ed. by G. V. Poore) ... ... ... ... ... ... ... ... ... 1883

**Duckworth, Sir D.** *A Treatise on Gout* ... ... ... ... ... ... ... ... ... 1890

**Eccles, A. S.** ... *Sciatica* ... ... ... ... ... ... ... ... ... ... ... ... 1893

**Ewald, Dr. C. A.** *Lectures on the Digestive Organs.* 3rd Ed. (Tr. by R. Saundby.) 2 vols. ... ... ... ... ... ... ... 1891-92

**Fenwick, E. Burckhardt and E. H.** *Atlas of Electric Cystoscopy* ... 1893

**Ferrier, D.**... ... *The Functions of the Brain* ... ... ... ... ... 2nd Ed. 1886

**Flügge, C.** ... ... *Micro-Organisms.* 2nd Ed. (Tr. by W. W. Cheyne) 1890

**Fothergill, J. M.** *Practitioner's Handbook of Treatment* ... ... 2nd Ed. 1880

Fox, W. ... ... *Atlas of the Pathological Anatomy of the Lungs.* (Ed. by W. R. Gowers) ... 1888

" ... ... *Diseases of the Lungs and Pleura.* (Ed. by S. Coupland) 1891

Galton, Sir D. ... *Healthy Hospitals* ... 1893

Garrod, A. E. ... *A Treatise on Rheumatism* ... 1890

*German Clinical Lectures* ... 1876

Gowers, W. R. ... *Diseases of the Nervous System.* 2nd Ed. 2 vols. 1892-93

Graves, R. J. ... *Clinical Medicine.* (Ed. by Dr. Neligan.) Vol. I. 1884

*Gynecology, Students' Handbook of* ... 1893

Harrison, R. ... *Surgical Disorders of the Urinary Organs* ... 4th Ed. 1893

Henoch, E.... ... *Lectures on Children's Diseases.* 4th Ed. (Tr. by J. Thomson)... Vol. I. 1889

*Higiene de la Educación* ... 1893

Jacobson, W. H. A. *The Operations of Surgery* ... 2nd Ed. 1891

Jenner, Sir W. ... *On Fevers and Diphtheria*, 1849 *to* 1879 ... 1893

Juler, H. E. ... *Ophthalmic Science and Practice* ... 2nd Ed. 1893

Koch, R. ... ... *Traumatic Infective Diseases.* (Tr. by W. W. Cheyne) 1880

Laveran, A. ... *Paludism.* (Tr. by J. W. Martin) ... 1893

Legg, J. W. ... *Examination of the Urine.* 7th Ed. (Ed. by H. L. Jones) ... 1893

Lehmann, K. B.... *Methods of Practical Hygiene.* (Tr. by W. Crookes.) 2 vols. ... 1893

Lewis, W. B. ... *A Text-book of Mental Diseases* ... 1889

Macdonald, G. ... *Diseases of the Nose* ... 2nd Ed. 1892

Maddox, E. E. ... *The Clinical Use of Prisms* ... (6) 2nd Ed. 1893

Marie and Dr. Souza-Leite, P. *Essays on Acromegaly* ... 1891

Martin, E.... ... *Essentials of Minor Surgery* ... 2nd Ed. 1893

Maule, J. Craig and W. *Medical Tracts for the Times.* 1st Series (1) 1886

Maunder, C. F. ... *Surgery of the Arteries* ... 1875

Metchnikoff, E.... *Comparative Pathology of Inflammation.* (Tr. by F. A. and E. H. Starling) ... 1893

Meyer, Dr. É. ... *A Practical Treatise on Diseases of the Eye.* (Tr. by F. Fergus) ... 1887

Muller, J. ... ... *Principles of Physics and Meteorology* ... (9) 1847

*Nomenclature of Diseases, The* ... (9) 2nd Ed. 1885

Parvin, T. ... ... *The Science and Art of Obstetrics* ... 2nd Ed. 1891

Peyrissac, Dr. E. *Étude sur les Abcès chroniques enkystés de l'Amygdale* 1893

Playfair, W. S.... *The Science and Practice of Midwifery.* 8th Ed. 2 vols. 1893

Poore, G. V. ... *Rural Hygiene* ... 1893

Power and L. W. Sedgwick, H. *Lexicon of Medicine and the Allied Sciences.* (New Syd. Soc.) 20 parts ... 1881-92

Pozzi, S. ... ... *A Treatise on Gynæcology.* 3 vols. ... 1892-93

*Psychological Medicine, Dictionary of.* (Ed. by D. H. Tuke.) 2 vols. ... 1892

Quekett, J.... ... *Lectures on Histology* ... (9) 1852

" ... ... *Practical Treatise on the Use of the Microscope* ... (9) 1848

Russell, Hon. R. *Epidemics, Plagues, and Fevers* ... 1892

Saint-Hilaire, I. G. *Histoire générale et particulière des anomalies de l'organisation* ... (9) 3 tomes 1832-36

Sedgwick, H. Power and L. W. *Lexicon of Medicine and the Allied Sciences.* (New Syd. Soc.) 20 parts ... 1881-92

*Selected Monographs* ... 1888

**Simpson, R.** ... *Sciatic Neuritis* ... 1893

**Smith, P.** ... *The Pathology and Treatment of Glaucoma* ... 1891

**Snow, H.** ... *On Cancers and the Cancer-Process* ... 1893

**Souza-Leite, P. Marie and Dr.** *Essays on Acromegaly* ... 1891

**Spiegelberg, O.** ... *A Text-book of Midwifery.* 2nd Ed. (Tr. by J. B. Hurry) ... Vol. II. 1888

**Squire, J. E.** ... *The Hygienic Prevention of Consumption* ... 1893

**Starr, M. A.** ... *Brain Surgery* ... 1893

**Stephenson, S.** ... *Ophthalmic Nursing* ... 1894

**Stokes, W.** ... *Diseases of the Chest.* (Ed. by A. Hudson) ... 1882

**Sutton, J. B.** ... *Tumours, Innocent and Malignant* ... 1893

*Therapeutics, A System of Practical.* (Ed. by H. A. Hare and W. Chrystie.) 3 vols. ... 1892

**Thorburn, W.** ... *Surgery of the Spinal Cord* ... 1889

**Trousseau, A.** ... *Clinical Medicine.* 3rd Ed. (Tr. by Sir J. R. Cormack) ... Vol. V. 1872

**Webster, J. C.** ... *Tubo-Peritoneal Ectopic Gestation* ... 1892

**White, W. H.** ... *Text-book of General Therapeutics* ... 1889

**Williams, C. J. B. and C. T.** *Pulmonary Consumption* ... 2nd Ed. 1887

**Wilson, A. M.** ... *Myxœdema* ... 1894

**Wright, H. Ashby and G. A.** *The Diseases of Children* ... 2nd Ed. 1892

**Yeo, I. B.** ... *A Manual of Medical Treatment.* 2 vols. ... 1893

### TRANSACTIONS, REPORTS, JOURNALS, &c.

American Journal of the Medical Sciences, The. (4) Vol. LXXIX., 1880; LXXX., 1880; (6) LXXXVI., 1883; (6) LXXXVIII.—XC. 1884-85

Annual of the Universal Medical Sciences. 5 vols. ... 1893

Asclepiad, The ... Vol. VII. 1890

Bibliotheca Medico-Chirurgica ... 1892

Calendar of the Royal College of Surgeons of England ... (2) 1871

Calendar of University College, Bristol ... (8) 1893

Clinical Journal, The ... Vol. II. 1893

Clinical Society's Transactions. (3) Vols. VIII., IX., 1875–76; (3) XI., 1878; XXVI. 1893

Hospitals—

St. Mary's Hospital—Registrar's Reports ... (5) 1879

St. Thomas's Hospital Reports ... Vol. XXI. 1893

International Journal of the Medical Sciences, The. (6) Vols. XCI.—CIV. 1886-92

Journal of the American Medical Association, The ... Vol. XX. 1893

Library Association Year-book for 1893, The ... 2nd Ed. 1893

Local Government Board, Tenth Annual Report of the ... (7) 1882

Medical Chronicle, The ... Vol. XVIII. 1893

Medical Review ... Vol. XXVII. 1893

St. Louis Medical and Surgical Journal, The ... Vol. LXIV. 1893

### LIST OF SUBSCRIBERS

TO

### The Bristol Medico-Chirurgical Journal.

*DECEMBER*, 1893.

---

### England.

### CHESHIRE.

CHESTER.
R. E. Burges

EGREMONT.

T. W. A. Napier

MACCLESFIELD.
W. R. Etches

### CORNWALL.

NEW QUAY.
A. Hardwick

PADSTOW.
H. F. Marley

PENZANCE.

W. S. Bennett

J. A. Fox

J. Symons

POLRUAN.
W. H. Torbock

PORT ISAAC.
R. J. George

ROCHE.

W. R. Goodfellow

ST. AUSTELL.
W. Mason

ST. JUST.
R. B. Searle

TORPOINT.
S. G. Vinter

### DEVONSHIRE.

BABBACOMBE.
T. Finch
G. F. P. Pizey

BAMPTON.
T. A. Guinness

BARNSTAPLE.
C. H. Gamble
J. Harper
M. Jackson
W. A. G. Laing
F. Penny

BRIXHAM.
G. C. Searle

BROAD CLYST.

J. W. Sandoe

J. Somer

BUDLEIGH SALTERTON.
H. F. Semple

CHAGFORD.
A. D. Hunt

CHERITON FITZPAINE.
G. L. Thorne

CHUDLEIGH.
C. L. Cunningham

DAWLISH.

A. de W. Baker

DEVONPORT.
F. E. Row

EXETER.
J. M. Ackland
A. G. Blomfield
H. Davy
P. M. Deas
E. J. Domville
W. Gordon
A. W. Kempe
E. B. Steele-Perkins
L. H. Tosswill

HONITON.
T. W. Shortridge

MILLBROOK.
A. B. Cheves

NEWTON ABBOT.
E. Haydon

### DEVONSHIRE *(Continued)*.

PAIGNTON.

J. Alexander

PLYMOUTH.

G. F. Aldous
R. H. Clay
E. L. Fox
R. H. Lucy
Medical Society
E. B. Thomson
W. L. Woollcombe

PLYMPTON.

C. Aldridge
W. D. Stamp

ST. MARY CHURCH.

T. Finch

TORQUAY.

W. Odell
R. Pollard
W. Powell
C. H. Wade

UFFCULME.

F. Morgan
B. G. Neale

WINKLEIGH.

J. H. Norman

YELVERTON.

A. P. Prowse

### DORSETSHIRE.

BEAMINSTER.

T. P. Daniel
F. P. Kitson

DORCHESTER.

F. B. Fisher
P. W. Macdonald

GILLINGHAM.

E. O. Cox

POOLE.

J. McNicoll

PORTLAND.

G. H. Lilley

STURMINSTER NEWTON

F. H. Hudson
J. C. Leach

WEYMOUTH.

R. W. Carter

J. M. Lawrie

WIMBORNE.

A. J. H. Crespi

### ESSEX.

NEWPORT.

W. A. Smith

### GLOUCESTERSHIRE.

BLAKENEY.

A. M. Irwin

BRISTOL.

W. R. Ackland
G. Adams
J. B. Adams
G. F. Atchley
W. M. Barclay
T. G. L. Baretti
B. J. Baron
A. E. Blacker
E Blacker
A. F. Blagg
E. C. Board
J. G. Boyce
J. Broom
E. F. H. Burroughs
J. P. Bush
J. Cameron
A. Carr
T. M. Carter
W. F. Carter
J. M. Clarke
E. W. Coathupe
R. W. Coe
T. V. Coker
T. J. Colman
H. Cook
G. G. Corbould
W. Cotton
F. R. Cross
J. Dacre
D. S. Davies
H. F. Devis
H. R. Dew
N. C. Dobson
W. Dowson
E. L. W. Dunbar
F. H. Edgeworth
W. Elder
C. Elliott
J. Ewens
R. G. Fendick
H. Fenton
J. L. Firth
T. Fisher
E. L. Fox
W. J. Fyffe
A. N. G. Gibbs
J. Gill
G. A. Gloag
W. C. Goodden
W. G. Grace
C. A. Griffiths
J. S. Griffiths
L. M. Griffiths
C. D. G. Hailes
A. H. Haines
A. J. Harrison
W. H. Harsant
A. G. Hayman
C. A. Hayman
J. C. Heaven
C. K. C. Herapath
H. E. Hetling
H. Hill
F. F. Hills
A. W. W. Hoffman
General Hospital
G. A. Imlay
Royal Infirmary
A. Jamison
D. G. Johnstcn
T. B. Kelly
F. P. Lansdown
R. G. P. Lansdown
A. E. A. Lawrence
H. Lawrence
E. L. Lees
Museum and Library
W. E. Lloyd
F. T. B. Logan
H. W. G. Macleod
W. T. Maddison
H. Marshall
C. E. Matthews
T. O. Mayor
J. S. Metford
J. Milne
H. F. Mole
C. A. Morton
G. T. Myles
W. N. Nevill
C. Newman
W. H. C. Newnham
J. Newstead
T. D. Nicholson
J. A. Norton
A. Ogilvy
G. S. Page
G. Parker
J. H. Parry
G. C. P. Pauli
W. J. Penny
C. F. Pickering
W. E. Pountney
J. J. Powell
A. Prichard
A. W. Prichard
J. E. Prichard
A. Pring

### GLOUCESTERSHIRE *(Continued).*

BRISTOL *(continued).*

A. B. Prowse
B. M. H. Rogers
W. B. Roué
C. K. Rudge
H. T. Rudge
J. E. Shaw
E. J. Sheppard
W. E. Shorland
D. Sinclair
E. M. Skerritt
G. M. Smith
J. G. Smith
R. Smith
R. S. Smith
R. V. Solly
C. Steele
W. H. Stevens
W. M. Stevens
F. W. Stoddart
J. Swain
J. G. Swayne
S. H. Swayne
W. C. Swayne
J. Taylor
W. J. Tivy
E. N. Tribe
H. Waldo
C. H. Walker
C. H. Wallace
J. H. Wathen
T. Webster
J. Wilding
P. W. Williams
H. W. Windsor-Aubrey
C. Wintle

CHELTENHAM.

Cheltenham Library
A. Duke
C. E. F. Mouat-Biggs
G. A. Peake
L. Winterbotham

CHIPPING SODBURY.

A. Grace

CINDERFORD.

W. C. Heane

CIRENCESTER.

W. R. Cossham

COLEFORD.

L. B. Trotter

DOWNEND.

H. Skelton

FISHPONDS.

W. Brown

GLOUCESTER.

R. W. Batten
T. Edis
J. G. Soutar

HAMBROOK.

E. Crossman
W. F. B. Eadon

KINGSWOOD.

H. Grace

LYDNEY.

W. P. Kennedy

MORETON-IN-MARSH.

L. K. Yelf

PARKEND.

W. C. Halpin

REDFIELD.

J. W. Rodgers

STAPLE HILL.

W. Murray

STAPLETON.

H. T. S. Aveline
H. A. Benham

STOKE BISHOP.

W. J. K. Millard
C. E. Wedmore

STONEHOUSE.

G. Carolin
G. T. B. Watters

STOW-ON-THE-WOLD.

E. Dening

STROUD.

A. S. Cooke
E. A. Howsin
F. W. Storry

TETBURY.

W. Wickham

THORNBURY.

E. M. Grace
T. H. Taylor
L. H. Williams

WESTBURY-ON-TRYM.

W. Duncan
H. Ormerod

WINTERBOURNE.

R. Eager

WOTTON-UNDER-EDGE.

D. H. Forty

### HAMPSHIRE.

ALDERSHOT.

J. C. Barr

ALTON.

W. L. P. Bevan

BOURNEMOUTH.

J. S. Dickie

D. C. Embleton

J. G. Harsant

J. Horsfall

A. J. Turner

CARISBROOKE.

J. Groves.

DROXFORD.

A. H. Goodwyn

FLEET.

H. Wilcox

LYMINGTON.

J. Rendall

ST. MARY BOURNE.

A. W. Riddell

SOUTHAMPTON.

W. R. Y. Ives

SOUTHSEA.

J. W. Cousins

VENTNOR.

R. Robertson
E. R. Woodford

### HEREFORDSHIRE.

HEREFORD.

W. C. Whitfeld

### HERTFORDSHIRE.

ST. ALBANS.

A. H. Boys

### KENT.

MAIDSTONE.

R. H. Norgate

TUNBRIDGE WELLS.

C. Wilson

### LANCASHIRE.

LIVERPOOL.

G. A. Hawkins-Ambler
Medical Institution
C. Williams

POULTON-LE-FYLDE.

P. H. Day

### MIDDLESEX.

LONDON.

A. E. Barker
J. Berry
L. Browne
J. G. Davey
J. F. Evans
A. P. Gould
J. M. Granville
G. W. Grote
M. Handfield-Jones
W. H. A. Jacobson
H. M. Jones
T. L. Laxton
J. Lister
E. J. Maclean
J. Macready
A. L. Marshall
W. Pippette
R. D. Powell
M. A. Ruffer
W. J. Seward
N. Smith
J. A. Watson
F. J. Wethered
G. S. Woodhead

TOTTENHAM.

W. H. Plaister

### MONMOUTHSHIRE.

BLAENAVON.

A. B. Avarne.

NEWPORT.

W. Basset

C. B. Gratte

H. R. Hudson

T. Limbery

O. E. B. Marsh

A. G. Thomas

J. T. Thomas

H. E. Williams

PONTYPOOL.

S. B. Mason

RHYMNEY.

T. H. Redwood

RISCA.

[illegible] L. [illegible] Robathan

TREDEGAR.

G. A. Brown

### NOTTINGHAMSHIRE.

NOTTINGHAM.

C. B. Taylor
W. H. Walter

### OXFORDSHIRE.

HENLEY-ON-THAMES.

J. Lidderdale

MIDDLETON CHENEY.

E. H. Openshaw

OXFORD.

R W. Doyne
H. P. Symonds

### SOMERSETSHIRE.

BATH.

G. A. Bannatyne
G. E. Bloxam
A. B. Brabazon
C. S. W. Cobbold
T. Cole
F. S. Cowan
S. Craddock
W. M. Ellis
A. E. W. Fox
H. W. Freeman
H. F. A. Goodridge
T. B. Goss
F. P. Hoblyn
H. C. Hopkins
L. J. King
F. Lace
H. Lane
T. P. Lowe
R. J. H. Scott
J. K. Spender
L. H. Walsh
L. A. Weatherly

BECKINGTON.

W. G. Evans

BRIDGWATER.

F. J. C. Parsons
J. B. Sincock

BRISLINGTON.

B. B. Fox
C. H. Fox

BURNHAM.

A. W. C. Peskett

CHEDDAR.

R. W. Statham

CHEW MAGNA.

E. T. Hale

CHILTON POLDEN.

G. Campbell

CLEVEDON.

T. Davis

J. A. F. Sawyer

S. Skinner

CREWKERNE.

E. J. Cave
W. W. Webber

CURRY RIVEL.

R. H. Vereker

### SOMERSETSHIRE *(Continued)*.

DULVERTON.
G. F. Sydenham

FRESHFORD.
C. E. S. Flemming

FROME.
F. Parsons
J. M. Rattray

GLASTONBURY.

J. A. Bright

HIGHBRIDGE.
R. Wade

ILMINSTER.
C. Munden

KEYNSHAM.
C. Harrison
G. G. D. Willett

LANGPORT.
J. Morgan

MIDSOMER N[illegible]TON.
A. W[illegible]l
A. H. Whicher

MILVERTON.
C. Randolph

NORTH PETHERTON.
C. F. Hawkins

PILL.
R. A. Ross

PORTISHEAD.
W. Monckton
C. A. Wigan

SHEPTON MALLET.
J. T. Hyatt

SOUTH PETHERTON.

S. W. MacIlwaine

TAUNTON.

H. J. Alford

Taunton Hospital

J. A. Macdonald

TEMPLE CLOUD.
T. Martin

THORNCOMBE.
F. A. Hill

TWERTON-ON-AVON.
J. Wigmore

WEDMORE.
J. Ford
R. P. Tyley

WELLINGTON.
J. Meredith

WELLS.

F. J. B. Bateman

W. Fairbanks

A. L. Wade

WESTON-SUPER-MARE

G. E. Alford

C. P. Crouch

G. B. Fraser

T. C. Grey

E. F. Martin

W. H. G. Phelps

G. F. Rossiter

R. Roxburgh

WORLE.
F. St. J. Kemm

WRINGTON.
A. H. Benson
H. W. Collins

YEOVIL.
P. S. H. Colmer

### STAFFORDSHIRE.

STAFFORD.
J. Scott

### SURREY.

EPSOM.
R. Davis

ESHER.
J. B. Fry

REDHILL.
C. M. Jessop

### SUSSEX.

BRIGHTON.
H. H. Tomkins

ST. LEONARD'S-ON-SEA.
W. H. Spencer

### WARWICKSHIRE.

BIRMINGHAM.
L. Tait

### WILTSHIRE.

BRADFORD-ON-AVON.
J. Beddoe

BURBAGE.
A. E. N. Browne

CALNE.
W. S. Batten
W. A. Hayes

DEVIZES.
T. B. Anstie
C. Eddowes
A. M. Gray

FOVANT.
C. Clay

MALMESBURY.
R. Kinneir

MERE.
J. McElfatrick

PEWSEY.
H. Colman

SALISBURY.
H. Coates
L. S. Luckham
H. J. Manning

SWINDON.
E. D. Duffett
H. W. M. Hayes
W. Howse
J. C. Maclean

WARMINSTER.
R. L. Willcox

### YORKSHIRE.

HARROGATE.
A. O. Jones

HULL.
J. B. Close

SHEFFIELD.
S. Snell

### Ireland.

### ANTRIM.

BELFAST.

J. W. Browne

J. A. Lindsay

### Scotland.

### EDINBURGHSHIRE.

EDINBURGH.
J. W. Ballantyne
Y. J. Pentland

### Wales.

### BRECONSHIRE.

BRECON.
A. Whyte

BRYNMAWR.
G. H. Browne

SENNY BRIDGE.
W. R. Jones

TALGARTH.
W. Howells

### CARDIGANSHIRE.

NEW QUAY.
E. R. Jones

### CARMARTHENSHIRE.

CARMARTHEN.
G. J. Hearder
R. G. Price

FERRYSIDE.
P. Williams

LLANELLY.
S. J. Roderick
D. J. Williams

### CARNARVONSHIRE.

BETHESDA.
J. William

### GLAMORGANSHIRE.

ABERAMAN.
J. B. James

ABERDARE.
E. Jones

CAERPHILLY.
J. Lewellyn

CARDIFF.

W. T. Edwards

J. Milward

D. R. Paterson

J. A. Phillips

W. Price

A. Rees

A. Sheen

Medical Society

J. L. Thomas

H. R. Vachell

COWBRIDGE.
T. R. Hamlen-Williams

DERI.
D. Kent-Jones

LLANDAFF.
J. Arthur

MAESTEG.
W. H. Thomas

MERTHYR TYDVIL.

W. W. Jones

C. E. G. Simons

J. L. W. Ward

PENARTH.
R. F. Nell

PORTH.
H. N. Davies

SWANSEA.

W. F. Brook

E. Davies

R. C. Elsworth

TREHARRIS.

W. W. Leigh

### PEMBROKESHIRE.

FISHGUARD.
H. L. Swete

HAVERFORDWEST.
E. P. Phillips

PENALLY.
B. C. Kendall

### Channel Islands.

GUERNSEY.
N. P. Webster

### Belgium.

COURTRAI.
Dr. Lauwers

### Greece.

CORFU.
Dr. Mavrojanni

### Italy.

GENOA.
B. Sebastiano

NAPLES.
C. Scimonelli

### Switzerland.

DAVOS-PLATZ.
W. R. Huggard

### Algeria.

ALGIERS.
W. Thomson

### Cape Colony.

FORT BEAUFORT.
J. Shaw

### United States.

COLORADO.
S. E. Solly

NEW YORK.
J. B. Jackson

### Victoria.

BRADFORD.
G. H. Skinner

### INDEX.

Abdominal hernia, Lectures on—W. H. Bennett (*Review*), 201

Adams, W.—Contractions of the fingers and hammer-toe (*Review*), 52.

Advertiser's A.B.C. (*Review*), 59.

Africa as a health-resort, South (*Review*), 57.

Alcohol and public health—Dr. J. J. Ridge (*Review*), 129.

Althaus, Dr. J.—Influenza (*Review*), 206.

American Surgical Association, Transactions of the (*Review*), 205.

Anæmia, Treatment of, 36.

Anæsthetics—Dr. D. W. Buxton (*Review*), 130.

Annual of the universal medical sciences (*Review*), 276.

Asthenopia and ocular headache—F. R. Cross, 73.

Atlas of clinical medicine—Dr. B. Bramwell (*Review*), 208.

Australasian medical directory (*Review*), 57.

Ballantyne, Dr. J. W.—The diseases and deformities of the fœtus (*Review*), 190.

Barclay, W. M.—Report on surgery, 38.

Barker, A. E.—A case of thrombosis of the femoral artery, 84.

Baron, Dr. B. J.—Reports on laryngology, 44, 187.

Bastian, Dr. H. C.—Hysterical or functional paralysis (*Review*), 196.

Bell, Dr. J.—Operations of surgery (*Review*), 131.

Bennett, W. H.—Lectures on abdominal hernia (*Review*), 201.

Bergonié et E. J. Moure, J.—Du traitement par l'électrolyse des déviations et éperons de la cloison du nez (*Review*), 56.

Blanc, Dr. H.—Advice to intending visitors to Cannes (*Review*), 209.

Blindness, Transitory, 263.

Blood and excreta, Clinical examination of the—Dr. S. Coupland (*Review*), 205.

Bourneville, Dr.—Recherches cliniques et thérapeutiques sur l'épilepsie, l'hystérie, et l'idiotie (*Review*), 54.
Boyce, Dr. R.—A text-book of morbid histology (*Review*), 50.
Bramwell, Dr. B.—Atlas of clinical medicine (*Review*), 208.
Branchial fistulæ, Two cases of—Dr. G. Parker, 89.
Bristol Eye Hospital, 142.
Bristol health report (*Review*), 209.
Bristol Medico-Chirurgical Society, 62, 135, 281.
Brodie, C. G.—Dissections illustrated (*Review*), 123.
Browning, Dr. W. W.—Modern homeopathy (*Review*), 274.
Bury, Dr. J. Ross and Dr. J. S.—On peripheral neuritis (*Review*), 192.
Bush, Dr. J. E. Shaw and J. P.—Lesion of the cauda equina, 161.
Buxton, Dr. D. W.—Anæsthetics (*Review*), 130.

Campbell, Dr. H.—Nervous organisation of man and woman (*Review*), 115.
Cancer of the rectum, 180.
Cannes, Advice to intending visitors to—Dr. H. Blanc (*Review*), 209.
Cardiac outlines—Dr. W. Ewart (*Review*), 118.
Carmichael, Dr. J.—Disease in children (*Review*), 126.
Carrington, Dr. R. E.—Notes on pathology (*Review*), 56.
Cartilages of knee, Dislocation of, 182.
Cathell, Dr. D. W.—Book on the physician (*Review*), 125.
Cauda equina, Lesion of the—Dr. J. E. Shaw and J. P. Bush, 161.
Cerna, Dr. D.—Notes on the newer remedies (*Review*), 194.
Cervical vertebræ, Acute inflammation around the upper—Dr. J. K. Spender, 1.
Chapman, Dr. H. C.—Medical jurisprudence and toxicology (*Review*), 194.

Charcot, Professeur—Clinique des maladies du système nerveux (*Review*), 116.
Cheltenham as a health-resort—Dr. J. H. Garrett, 241.
Chemistry, A manual of—Dr. A. P. Luff (*Review*), 117.
Children, Disease in—Dr. J. Carmichael (*Review*), 126.
Chlorosis, 103.
Clarke, Dr. E.—Eyestrain (*Review*), 123.
Clarke, Dr. J. M.—Report on medicine, 32.
Clinical Journal (*Review*), 57.
Cobbold, Dr. C. S. W.—The alleged increase of insanity, 145.
Coca and cocaine—W. Martindale (*Review*), 130.
Cocaine, Use of, 187.
Constant, T. E.—How to give gas (*Review*), 204.
Consumption, Antiseptic dry-air treatment of—Dr. J. J. Hartnett (*Review*), 203.
Cooper, F. S. Edwards and A.—Diseases of the rectum and anus (*Review*), 53.
Corneal ulcers, 264.
Coupland, Dr. S.—Examination of the blood and excreta (*Review*), 205.
Cozzolino, Dr. V.—The hygiene of the ear (*Review*), 207.
Cross, F. R.—Asthenopia and ocular headache, 73; Report on ophthalmology, 262.
Crosse, W. H.—Malarial fevers (*Review*), 205.

Davis, Dr. N. S.—Diseases of the lungs, heart, and kidneys (*Review*), 191.
Dermic memoranda—Dr. W. Gemmell (*Review*), 57.
Diabetes, 178.
Diagnosis, Physical—Dr. G. A. Gibson and Dr. W. Russell (*Review*), 209.
Dictionary of Medicine—Dr. J. M. Keating and H. Hamilton (*Review*), 128.
Diphtheria, Loeffler bacillus in, 188.
Dissections illustrated—C. G. Brodie (*Review*), 123.
Dixey, Dr. F. A.—Epidemic influenza (*Review*), 123.
Dowse, Dr. S.—Massage (*Review*), 129.

Ear, The hygiene of the—Dr. V. Cozzolino (*Review*), 207.
Edwards, A. Cooper and F. S.—Diseases of the rectum and anus (*Review*), 53.
Electricity, Medical—Dr. W. E. Steavenson and Dr. H. L. Jones (*Review*), 54.
Eminson, T. B. F.—Epidemic pneumonia at Scotter (*Review*), 129.
Empyema, A case of double—Dr. W. J. Fyffe, 233.
English illustrated magazine (*Review*), 277.
Enteric fever, 100.
Epiglottis, 44.
Épilepsie, l'hystérie, et l'idiotie—Dr. Bourneville (*Review*), 54.
Ewart, Dr. W.—Cardiac outlines (*Review*), 118; Symptoms and physical signs (*Review*), 199.
Eye, Diseases of the—Dr. G. E. de Schweinitz (*Review*), 121; Dr. H. R. Swanzy (*Review*), 131.
Eyesight, Effect of position on, 265.
Eyestrain—Dr. E. Clarke (*Review*), 123.

Farquharson, Dr. A. C.—Ptomaines and other animal alkaloids (*Review*), 55.

Fenwick, E. H.—Rules of surgical practice (*Review*), 132.

Fevers, 175.

Fingers and hammer-toe, Contractions of the—W. Adams (*Review*), 52.

Fistula-in-ano, 258.

Flat-foot, Treatment of, 40.

Fœtus, The diseases and deformities of the—Dr. J. W. Ballantyne (*Review*), 190.

Forceps delivery, Compression of the umbilical cord during—Dr. J. G. Swayne, 229.

Forsbrook, Dr. W. H. R.—A dissertation on osteo-arthritis (*Review*), 198.

Fractures, Over-riding fragments in oblique, 257.

Fry, Sir E.—Address to medical students, 267.

Fyffe, Dr. W. J.—A case of double empyema, 233.

Gall-stones and their treatment—A. W. M. Robson (*Review*), 51.

Gangrene, Thrombosis of the femoral artery leading to—A. E. Barker, 84.

Garrett, Dr. J. H.—Cheltenham as a health-resort, 241.

Gas, How to give—T. E. Constant (*Review*), 204.

Gemmell, Dr. W.—Dermic memoranda (*Review*), 57.
Gestation, A case of extra-uterine—Dr. A. E. A. Lawrence and C. A. Morton, 29.
Gibson, Dr. W. Russell and Dr. G. A.—Physical diagnosis (*Review*), 209.
Goodhart, Dr. J. F.—On common neuroses (*Review*), 53.
Gowers, Dr. W. R.—Syphilis and the nervous system (*Review*), 195.
Griffith, Dr. A. H.—Intra-ocular growths (*Review*), 272.
Griffiths, L. M.—Report on obstetrics, 108.

Hamilton, Dr. J. M. Keating and H.—Dictionary of medicine (*Review*), 128.

Harris, J. D.—Scab-healing (*Review*), 272.

Harrogate mineral waters—Dr. H. J. Johnston-Lavis (*Review*), 130.

Harsant, W. H.—Report on surgery, 104.

Hartnett, Dr. J. J.—Antiseptic dry-air treatment of consumption (*Review*), 203.

Haydon, F.—Ophthalmic atlas (*Review*), 206.

Health: voyage to South Africa and sojourn there (*Review*), 57.

Heart and thoracic aorta, The diagnosis of diseases of the—Dr. A. E. Sansom (*Review*), 113.

Heart disease in children, 37.

Hedley, Dr. W. S.—The hydro-electric methods in medicine (*Review*), 117.

Heredity in diseases of the eye, 264.

Heredity, One view of—A. Prichard, 91.

Hernia, Radical cure of, 104.

Hodgkins fund prizes, 141.

Homatropine in eye diseases, 265.

Homeopathy, Modern—Dr. W. W. Browning (*Review*), 274.

Hospital annual, Burdett's (*Review*), 272.

Hospital-ward to consulting-room, From (*Review*), 275.

Hydro-electric methods in medicine, The—Dr. W. S. Hedley (*Review*), 117.

Hygiene and public health—Dr. L. C. Parkes (*Review*), 131; Dr. B. A. Whitelegge (*Review*), 207.

Hypnotism in chronic alcoholism—Dr. C. L. Tuckey (*Review*), 204.

Hysterical or functional paralysis—Dr. H. C. Bastian (*Review*), 196.

Indian medico-chirurgical review (*Review*), 273.
Influenza, 44; Dr. J. Althaus (*Review*), 206; Dr. F. A. Dixey (*Review*), 123.
Insane, Reform in the treatment of the—Dr. D. H. Tuke (*Review*), 202.
Insanity, The alleged increase of—Dr. C. S. W. Cobbold, 145.
Intra-ocular growths—Dr. A. H. Griffith (*Review*), 272.
Ireland, Transactions of the Royal Academy of Medicine in (*Review*), 206.
Iritis, Short-sight in, 263.

Johns Hopkins hospital reports (*Review*), 199.
Johnston-Lavis, Dr. H. J.—Harrogate mineral waters (*Review*), 130.
Jones, H.—A guide to the examinations in sanitary science (*Review*), 131.
Jones, Dr. W. E. Steavenson and Dr. H. L.—Medical electricity (*Review*), 54.
Journalism, Modern medical—J. G. Smith, 217.
Jurisprudence and toxicology, Medical—Dr. H. C. Chapman (*Review*), 194.

Karkeek, P. Q.—Torquay as a health-resort, 93.
Keating and H. Hamilton, Dr. J. M.—Dictionary of medicine (*Review*), 128.
Kenwood, Dr. H. R.—Public health laboratory work (*Review*), 270.

Lane, H.—Differentiation in rheumatic disease (so called) (*Review*), 56.
"Lanoline," From "Œsypum" to (*Review*), 132.
Laryngology, Reports on—Dr. B. J. Baron, 44, 187.
Lateral curvature of the spine—N. Smith (*Review*), 207.
Lawrence and C. A. Morton, Dr. A. E. A.—A case of extra-uterine gestation, 29.
Leeward Islands medical journal (*Review*), 274.
Library of the Bristol Medico-Chirurgical Society, 64, 137, 212, 282.
Library of the Surgeon-General's Office, United States Army, Index-catalogue of (*Review*), 58.
Luff, Dr. A. P.—A manual of chemistry (*Review*), 117.

Lungs, heart, and kidneys — Dr. N. S. Davis (*Review*), 191.

Macready, J. F. C. H.—A treatise on ruptures (*Review*) 271.
Malarial fevers — W. H. Crosse (*Review*), 205.
Malignant disease, The medicinal treatment of, 176.
Manchester, Transactions of the Pathological Society of (*Review*), 56.
Martindale, W.—Coca and cocaine (*Review*), 130; The extra pharmacopœia (*Review*), 129.
Massage, A primer on the art of—Dr. S. Dowse (*Review*), 129.
Materia medica—Dr. W. H. White (*Review*), 51.
Medical annual (*Review*), 58.
Medical education, A history of—Dr. T. Puschmann (*Review*), 48.
Medicine, Practice of—Dr. A. A. Stevens (*Review*), 194.
Medicine, Reports on—Dr. J. M. Clarke, 32; Dr. G. Parker, 175; Dr. J. E. Shaw, 253; Dr. R. S. Smith, 100.
Mental diseases, Epitome of—Dr. J. Shaw (*Review*), 122.
Mercurial treatment, 185.
Meredith, Dr. J.—Rötheln, 171.
Microscopy, Medical—Dr. F. J. Wethered (*Review*), 118.
Middlesex hospital reports (*Review*), 207.
Miners' nystagmus — S. Snell (*Review*), 121.
Morbid histology, A text-book of—Dr. R. Boyce (*Review*), 50.
Morton, Dr. A. E. A. Lawrence and C. A.—A case of extra-uterine gestation, 29.
Moullin, C. W. M.—The operative treatment of enlargement of the prostate (*Review*), 120.
Moure, J. Bergonié et E. J.—Du traitement par l'électrolyse des déviations et éperons de la cloison du nez (*Review*), 56.
Mouth shut, Keep your—Dr. F. A. A. Smith (*Review*), 129.
Myxœdema, 32.

Nasal neuroses, 45.
Nasal stenosis, 189.
Nerveux, Clinique des maladies du système — Professeur Charcot (*Review*), 116.
Nervous organisation of man and woman — Dr. H. Campbell (*Review*), 115.
Neuritis, On peripheral — Dr. J. Ross and Dr. J. S. Bury (*Review*), 192.
Neuroses, On common—Dr. J. F. Goodhart (*Review*), 53.
Newman, Dr. D.—Malignant disease of the throat and nose (*Review*), 127.
Nez, Du traitement de la cloison du—J. Bergonié et E. J. Moure (*Review*), 56.
Nursing during lying-in period, 110.

Obstetric aphorisms — Dr. J. G. Swayne (*Review*), 276.
Obstetrics, Report on — L. M. Griffiths, 108.
Odell, W.—Recreation (*Review*), 275.
Ophthalmic atlas — F. Haydon (*Review*), 206.
Ophthalmology, Report on—F. R. Cross, 262.
Organic extracts in medicine, 253, 265.
Osteo-arthritis, A dissertation on—Dr. W. H. R. Forsbrook (*Review*), 198.
Ovaries and Fallopian tubes, Diseases of — J. B. Sutton (*Review*), 46.
Ovaries, tubes, and uterus, Conservative operations of the, 260.

Parker, Dr. G.—Two cases of branchial fistulæ, 89; Report on medicine, 175.

Parkes, Dr. L. C.—Hygiene and public health (*Review*), 131.

Patent alias quack medicines (*Review*), 207.

Pathology and bacteriology, Journal of (*Review*), 59, 208.

Pathology, Notes on — Dr. R. E. Carrington (*Review*), 56.

Penny, W. J.—Report on surgery, 180.

Pharmacopœia, The extra — W. Martindale (*Review*), 129.

Phthisis, Laryngeal, 189.

Physician, Book on the—Dr. D. W. Cathell (*Review*), 125.

Physicians', surgeons', and consultants' visiting list (*Review*), 277.

Physiology for students—Dr. A. T. Schofield (*Review*), 270.

Pilocarpine in rheumatic affections of the eye, 264.

Pleural effusions—Dr. A. Sheen, 14.

Plymouth medical society, 282.

Pneumonia at Scotter, Epidemic—T. B. F. Eminson (*Review*), 129.

Post-graduate, The (*Review*), 273.

Pregnancy, Examination during, 112.
Prichard, A.—One view of heredity, 91.
Prostate, The operative treatment of enlargement of the—C. W. M. Moullin (*Review*), 120.
Ptomaines and other animal alkaloids — Dr. A. C. Farquharson (*Review*), 55.
Public health laboratory work—Dr. H. R. Kenwood (*Review*), 270.
Puschmann, Dr. T.—A history of medical education (*Review*), 48.

Recreation—W. Odell (*Review*), 275.

Rectum and anus, Diseases of the —A. Cooper and F. S. Edwards (*Review*), 53.

Rectum, Cancer of the, 180.

Remedies, Notes on the newer—Dr. D. Cerna (*Review*), 194.

Retina, Treatment of detachment of the, 262.

Rheumatic disease (socalled), Differentiation in — H. Lane (*Review*), 56.

Rhinitis, Croupous, 45.

Ridge, Dr. J. J.—Alcohol and public health (*Review*), 129.

Robson, A. W. M.—On gall-stones and their treatment (*Review*), 51.

Rome and the Roman malaria—Professor Tommasi-Crudeli (*Review*), 124.

Ross, Dr. J. S. Bury and Dr. J.—On peripheral neuritis (*Review*), 192.

Rötheln—Dr. J. Meredith, 171.

Royal College of Physicians of Edinburgh, Reports from the laboratory of the (*Review*), 119.

Ruptures, A treatise on—J. F. C. H. Macready (*Review*), 271.

Russell, Dr. G. A. Gibson and Dr. W.—Physical diagnosis (*Review*), 209.

Saint Bartholomew's hospital reports (*Review*), 274.
Sanitary science, A guide to the examinations in—H. Jones (*Review*), 131.
Sansom, Dr. A. E.—The diagnosis of diseases of the heart and thoracic aorta (*Review*), 113.
Savill, Dr. T. D.—On an epidemic skin disease (*Review*), 127.
Scab-healing—J. D. Harris (*Review*), 272.
Scarlatina, 103.
Schofield, Dr. A. T.—Physiology for students (*Review*), 270.
Schweinitz, Dr. G. E. de—Diseases of the eye (*Review*), 121.
Scraps, 71, 143, 215, 287.
Shaw, Dr. J.—Epitome of mental diseases (*Review*), 122.
Shaw, Dr. J. E.—Report on medicine, 253.
Shaw, J. P. Bush and Dr. J. E.—Lesion of the cauda equina, 161.
Sheen, Dr. A.—Pleural effusions, 14.
Sick, Care of the—Florence Stacpoole (*Review*), 204.
Sick, Preparations for the, 60, 132, 210, 278.
Skin disease — Dr. T. D. Savill (*Review*), 127.
Smith, Dr. F. A. A.—Keep your mouth shut (*Review*), 129.
Smith, Dr. R. S.—Report on medicine, 100.
Smith, J. G. — Modern medical journalism, 217.
Smith, N.—Lateral curvature of the spine (*Review*), 207.
Snell, S. — Miners' nystagmus (*Review*), 121.
Spender, Dr. J. K.—Acute inflammation around the upper cervical vertebræ, 1.
Spinal abscesses, Treatment of, 38.
Stacpoole, Florence — Advice to women before, during, and after confinement (*Review*), 204; Our sick, and how to nurse them (*Review*), 204.
Steavenson, Dr. H. L. Jones and Dr. W. E.—Medical electricity (*Review*), 54.
Stevens, Dr. A. A.—Practice of medicine (*Review*), 194.
Surgery, Operations of—Dr. J. Bell (*Review*), 131.
Surgery, Reports on—W. M. Barclay, 38; W. H. Harsant, 104; W. J. Penny, 180; Dr. J. Swain, 257.
Surgical operations, The student's handbook of—F. Treves (*Review*), 52.
Surgical practice, Rules of—E. H. Fenwick (*Review*), 132.
Sutton, J. B. — Diseases of the ovaries and Fallopian tubes (*Review*), 46.
Swain, Dr. J.—Report on surgery, 257.
Swanzy, Dr. H. R.—Diseases of the eye (*Review*), 131.
Swayne, Dr. J. G.—Compression of the umbilical cord during forceps delivery, 229; Obstetric aphorisms (*Review*), 276.
Symphysiotomy, 108.

Symptoms and physical signs—Dr. W. Ewart (*Review*), 199.
Syphilis and the nervous system—Dr. W. R. Gowers (*Review*), 195.
Syphilis and tuberculosis, The combination of—Dr. P. W. Williams, 155.

Taylor, J.—Three cases of umbilical hemorrhage occurring in the same family, 237.
Temporal welfare (*Review*), 277.
Throat and nose, Malignant disease of the—Dr. D. Newman (*Review*), 127.
Thyroid gland, Treatment of myxœdema by injections of extract of the, 32.
Toe-nail, In-growing, 259.
Tommasi-Crudeli, Professor—The climate of Rome and the Roman malaria (*Review*), 124.
Torquay as a health-resort—P. Q. Karkeek, 93.
Transfusion in surgical practice, 42.
Treves, F.—The student's handbook of surgical operations (*Review*), 52.
Tuberculosis of the joints, 184.
Tuckey, Dr. C. L.—Hypnotism in chronic alcoholism (*Review*), 204.
Tuke, Dr. D. H.—Reform in the treatment of the insane (*Review*), 202.

Umbilical cord during forceps delivery, Compression of the—Dr. J. G. Swayne, 229.
Umbilical hemorrhage, Three cases of—J. Taylor, 237.
University college, Bristol: Faculty of Medicine, 266.
Urine, Chart for recording the examination of the (*Review*), 207.

Vitreous body, Antiseptic injections into the, 262.

Voice-production, The influence of faulty, 44.

Wethered, Dr. F. J. — Medical microscopy (*Review*), 118.
White, Dr. W. H.—Materia medica (*Review*), 51.
Whitelegge, Dr. B. A.—Hygiene and public health (*Review*), 207.
Williams, Dr. P. W.—The combination of syphilis and tuberculosis, especially in regard to laryngeal affections, 155.
Women, Advice to—Florence Stacpoole (*Review*), 204.

Year-book of scientific societies (*Review*), 273.
Year-book of treatment (*Review*), 58.

### PUBLICATIONS RECEIVED.

From BAILLIÈRE, TINDALL & COX, London:
*Morbid Growths and Sporozoa.* By J. JACKSON CLARKE, M.B. 1893.
*Aids to the Treatment of Diseases of Children.* By JOHN MCCAW, M.D. [1893.]
*Aids to Otology.* By W. R. H. STEWART. [1893.]
*Diphtheria and its Treatment.* By BROWNLOW R. MARTIN, M.B. 1894.

From JOHN BALE & SONS, London:
*Syphilis.* By EDWARD COTTERELL. 1893.
*The Nerve Theory of Menstruation.* By CHRISTOPHER MARTIN, M.B. 1893.

From CASSELL AND COMPANY, LIMITED, London:
*A Manual of Medical Treatment or Clinical Therapeutics.* 2 vols. By J. BURNEY YEO, M.D. 1893.
*Tumours, Innocent and Malignant.* By J. BLAND SUTTON. 1893.
*Diseases of the Skin.* By MALCOLM MORRIS. 1894.

From J. & A. CHURCHILL, London:
*Surgical Diseases of the Urinary Organs.* By REGINALD HARRISON. Fourth Edition. 1893.
*On Cancers and the Cancer Process.* By HERBERT SNOW, M.D. 1893.

From DAVID CLAPP & SON, Boston:
*Tuberculous Pleurisy.* By WILLIAM OSLER, M.D. 1893. [Reprint.]

From THE CLARENDON PRESS, Oxford
*Healthy Hospitals.* By Sir DOUGLAS GALTON. 1893.

From CORDES, HERMANNI & CO., Hamburg:

*Ueber die antiseptische Kraft des Ichthyols.* Von Dr. RUDOLF ABEL. 1893. [Reprint.]

*Über Ichthyolsuppositorien bei der Behandlung der Prostatitis.* Von Dr. A. FREUDENBERG. 1893. [Reprint.]

Dott. REMO SEGRÈ, *L' Ittiolo nella terapia delle forme cutanee e venereo-sifilitiche.* 1893. [Reprint.]

*L'Ictiolo nella cura della blenorragia.* Dott. PIO COLOMBINI. 1893. [Reprint.]

From DAMRELL & UPHAM, Boston:
*Physic and Physicians as Depicted in Plato.* By WILLIAM OSLER, M.D. 1893. [Reprint.]
*Four Successful Nephrectomies.* By MAURICE H. RICHARDSON, M.D. 1893. [Reprint.]

From Professor SAMUEL G. DIXON, M.D., Philadelphia:
*Address on Hygiene.* Delivered by Prof. SAMUEL G. DIXON, M.D. [N.D.]
*The Bile Salts, Urea, etc., as Therapeutic Agents.* By SAMUEL G. DIXON, M.D. 1893. [Reprint.]

From OCTAVE DOIN, Paris:

*Étude sur les Abcès chroniques enkystés de l'Amygdale.* Par le Dr. EUG. PEYRISSAC. 1893.

From W. A. DUNKERLEY, London:
*To-day.* No. 1. November 11th, 1893.

From GEORGE M. GOULD, A.M., M.D., Philadelphia:
*The Pernicious Influence of Albinism upon the Eye.* By GEORGE M. GOULD, M.D. 1893. [Reprint.]
*The Duty of the Community to Medical Science.* By GEORGE M. GOULD, M.D. 1893. [Reprint.]

From George M. Gould, A.M., M.D., Philadelphia:
*The Medical Press.* By George M. Gould, M.D. 1893. [Reprint.]
*The Meaning and the Method of Life.* By George M. Gould, A.M., M.D. Reviewed by Josiah Royce. 1893. [Reprint.]

From Charles Griffin & Company, Limited, London:
*The Hygienic Prevention of Consumption.* By J. Edward Squire, M.D. 1893.

From Henrich y Ca., Barcelona:

*Higiene de la Educación.* 1893.

From M. Henry, London:
*Food and Sanitation.* Vol. III., No. 67. November 18th, 1893.

From Ingram Bros., London:
*The English Illustrated Magazine.* Vol. XI., Nos. 121–2. October, November, 1893.

From H. K. Lewis, London:
*Examination of the Urine.* By J. Wickham Legg. Seventh Edition. Edited and Revised by H. Lewis Jones, M.D. 1893.
*A Guide to the Public Medical Services.* By Alexander S. Faulkner. 1893.

From E. & S. Livingstone, Edinburgh:
*The Students' Handbook of Gynecology.* 1893.

From Macmillan & Co., London:
*Sciatica.* By A. Symons Eccles, M.B. 1893.
*A Text-book of General Therapeutics.* By W. Hale White, M.D. 1889.

From Dr. G. de Maurans, Paris:
*The Medical Week.* Vol. I., No. 49. December 8th, 1893.

From The National Temperance League Publication Depot, London:
*The Medical Pioneer.* Sept.—Nov., 1893.

From John Morgan Richards, London;
*Medical Reprints.* Sept.—Nov., 1893.

From W. P. Saunders, Philadelphia;
*Essentials of Minor Surgery.* By Edward Martin, M.D. Second Edition. 1893.

From The Scientific Press, Limited, London:
*Ophthalmic Nursing.* By Sydney Stephenson, M.B. 1894.
*Myxœdema.* By A. Marius Wilson, M.D. 1894.

From David Stott, London:

*The Stœchiological Cure of Consumption.* Third Edition. By J. F. Churchill, M.D. 1893.

From Dottor Francesco Villardi, Milano:
*Raro Caso di Empiema pulsatile.* Pel Dott. Emilio Pittarelli. 1893. [Reprint.]

From Ward, Lock & Bowden, Limited, London:
*Sylvia's Home Journal.* Christmas Number. 1893.

From Whittaker & Co., London:
*Dissections Illustrated.* By C. Gordon Brodie. Part II. [1893.]

From Wiebeck y Turtl, Buenos Ayres:

*Revista de la Soriedad Moenia Argentina.* Vol. II., No. 10. 1893.

From John Wright & Co., Bristol:

*Ophthalmological Prisms.* By Ernest E. Maddox, M.D. Second Edition. 1893.

*Sciatic Neuritis.* By Robert Simpson. 1893.

